# Targeting cellular mRNAs translation by CRISPR-Cas9

**DOI:** 10.1038/srep29652

**Published:** 2016-07-13

**Authors:** Yuchen Liu, Zhicong Chen, Anbang He, Yonghao Zhan, Jianfa Li, Li Liu, Hanwei Wu, Chengle Zhuang, Junhao Lin, Qiaoxia Zhang, Weiren Huang

**Affiliations:** 1 Key Laboratory of Medical Reprogramming Technology, Shenzhen Second People’s Hospital, First Affiliated Hospital of Shenzhen University, Shenzhen 518039, China; 2 Department of Urological Surgery, Shenzhen Second People’s Hospital, The First Affiliated Hospital of Shenzhen University, Shenzhen 518039, China

## Abstract

Recently CRISPR-Cas9 system has been reported to be capable of targeting a viral RNA, and this phenomenon thus raises an interesting question of whether Cas9 can also influence translation of cellular mRNAs. Here, we show that both natural and catalytically dead Cas9 can repress mRNA translation of cellular genes, and that only the first 14 nt in the 5′ end of sgRNA is essential for this process. CRISPR-Cas9 can suppress the protein expression of an unintended target gene without affecting its DNA sequence and causes unexpected phenotypic changes. Using the designed RNA aptamer-ligand complexes which physically obstruct translation machinery, we indicate that roadblock mechanism is responsible for this phenomenon. Our work suggests that studies on Cas9 should avoid the potential off-target effects by detecting the alteration of genes at both the DNA and protein levels.

The bacterial type II CRISPR-Cas9 system is emerging as a versatile tool for basic biology and engineering biology^1^,^2^,^3^,^4^,^5^. The Cas9 endonuclease can target and cleave double –stranded DNA containing protospacer adjacent motif (PAM) (NGG, N indicates any base) through a single guide RNA (sgRNA)^6^. The specificity of DNA targeting is determined by both sgRNA-DNA base pairing and PAM sequence. CRISPR - Cas9 is usually reprogrammed to mediate genome modifications in prokaryotic and eukaryotic systems and has great applications in industry, agriculture and medicine^7^,^8^. Nuclease-deficient Cas9 fused with transcriptional activators or repressors is able to activate or inactivate transcription of the target genes on a genome-wide scale^9^,^10^,^11^. Cas9 can also cleave single-stranded DNA/RNA targets when the PAM-presenting oligonucleotides are presented as separate DNA^12^,^13^. Despite broad interest in genome editing and gene regulation, this technology is limited by the problems of off-target effects^14^,^15^,^16^. Specific guidelines for reducing these effects are being developed based on the results of deep sequencing^16^,^17^,^18^,^19^.

In contrast to the DNA interference induced by CRISPR-Cas9, the traditional technologies —including antisense RNAs, ribozymes, and RNA interference — influence gene expression at the level of mRNA^20^,^21^,^22^. Antisense RNAs directly inhibit translation of a complementary mRNA by Watson-Crick base pairing. In the design of a reprogrammed ribozyme, a guide sequence is used to hybridize to the target mRNA and guide ribozyme for the specific cleavage. Small interfering RNAs, such as siRNAs, shRNAs and microRNAs, mediate gene silencing by directing the RNA-induced silencing complex (RISC) to bind to and degrade the mRNA. Based on these observations that mRNA target can be bound by a separate sequence that is complementary to mRNA, and that the sgRNA also has an antisense element, we wondered whether the sgRNA-Cas9 complex can affect the mRNA stability or translation. While the sgRNA of other CRISPR-Cas systems can target RNA^23^, Cas9 has been thought to be incapable of cleaving RNA without the presence of a separate PAM-presenting oligonucleotide^13^. Until recently, it has been reported that the sgRNA-Cas9 complexes can bind to the RNA of hepatitis C virus (HCV) independent of PAM and inhibit viral protein production^24^. Although eukaryotic ribosome can translate both cellular mRNAs and viral RNAs, their mechanisms of translation initiation are completely different. The translation of HCV RNA is mediated by internal ribosome entry site (IRES), whereas translation of cellular mRNAs is triggered by the 5′-cap structure containing multiple initiation factors. Therefore, it is still unclear whether sgRNA-Cas9 complex can affect mRNA translation of cellular genes in the absence of PAM-carrying DNA.

In this study, we investigated the effect of sgRNA-Cas9 complex on mRNA translation of cellular genes in human cells and our data revealed that this protein has an additional and yet un-described activity for repression of cellular mRNA translation.

## Results

### Wild-type Cas9 reduces luciferase activity through inhibition of its translation

**Figure 1:**
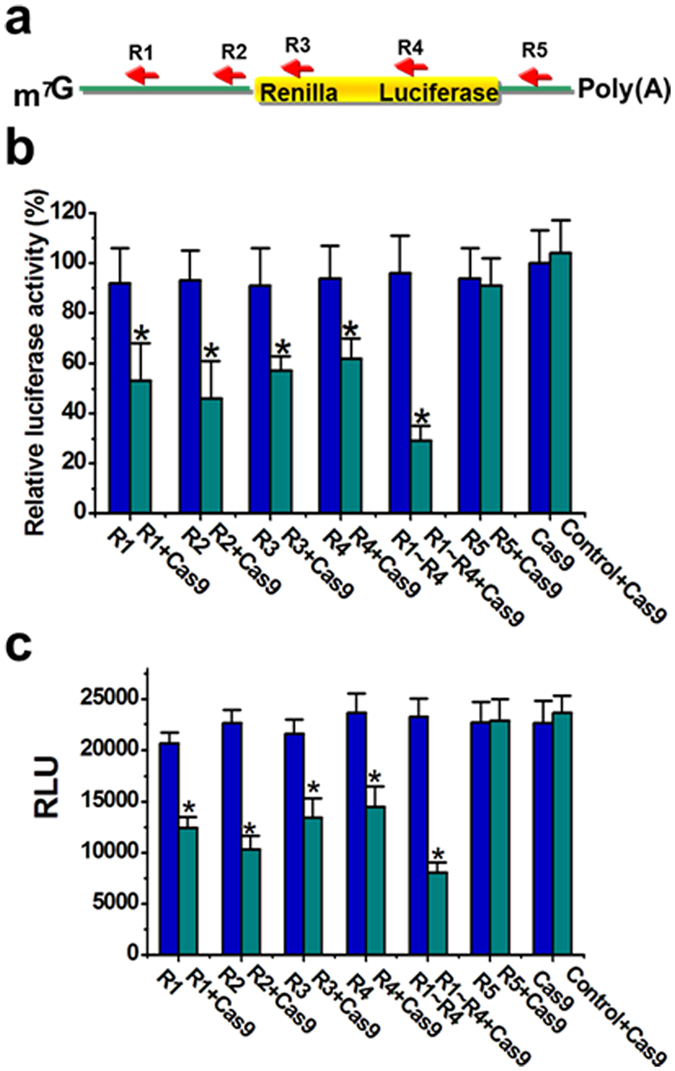
CRISPR-Cas9 suppressed activity of luciferase both *in vivo* and *in vitro*. (**a**) Design of the sgRNAs targeting various regions of Renilla luciferase mRNA. (**b**) Suppression of luciferase expression was achieved by each sgRNA (R1, R2, R3 or R4) combined with wild-type Cas9 protein in Hela cells. Introduction of either the sgRNA or Cas9 alone had no such effect, nor did introduction of a nonspecific sgRNA control and Cas9. Reported data were shown as mean ± SD from three biological replicates. *P < 0.05 compared to nonspecific sgRNA control by paired, one-sided t-test. (**c**) Suppression of luciferase expression was achieved by each sgRNA (R1, R2, R3 or R4) combined with wild-type Cas9 protein *in vitro*, while other groups had no such effects. Reported data were shown as mean ± SD from three biological replicates. *P < 0.05 compared to nonspecific sgRNA control by paired, one-sided t-test.

We designed 5 sgRNAs (R1, R2, R3, R4 and R5) complementary to different regions of the mRNA of Renilla luciferase gene encoded on psiCHECK™-2 vector, either binding to untranslated regions (UTRs) or to the coding sequences (Fig. 1a and Supplementary Table 1). The common feature of the corresponding DNA sequences of the targeted regions is that they all lack the PAM sequences. We used this strategy to shut-off the possible DNA interference effects. psiCHECK™-2 was cotransfected into Hela cells with wild-type catalytically active Cas9 (S. pyogenes) containing the nuclear localization signal (NLS), and luciferase reporter assays were conducted. We also confirmed that the subcellular localization for Cas9-NLS is nucleus (Supplementary Fig. 1). Except for R5, introduction of individual sgRNAs modestly decreased luciferase expression in the presence of Cas9 protein (Fig. 1b). Multiple sgRNAs (R1∼R4) acted synergistically to induce robust luciferase inhibition. To exclude the possibility that the sgRNA/Cas9 complexes could also cleave the non-canonical PAM sequences of DNA and reduce luciferase expression, we performed an *in vitro* translation reaction using purified wild-type Cas9 protein, *in vitro* transcribed sgRNA, as well as *in vitro* transcribed mRNA of Renilla luciferase. All the sgRNAs except R5 decreased translation of the Renilla luciferase to some extent after addition of Cas9 protein, as measured by luciferase activity (Fig. 1c). Stronger repression was achieved when the combination of sgRNAs (R1 ∼ R4) was used in the experiment. Moreover, to further confirm that mRNA is also being targeted *in vivo*, we also transfected a version of wild-type Cas9 lacking the NLS into the Hela cells harboring psiCHECK™-2 vector, and similar results were observed (Supplementary Fig. 2). These data indicated that sgRNA-Cas9 complex could directly inhibit translation of mRNA of Renilla luciferase.

### Characterization of the mismatches that influence silencing efficiency

**Figure 2:**
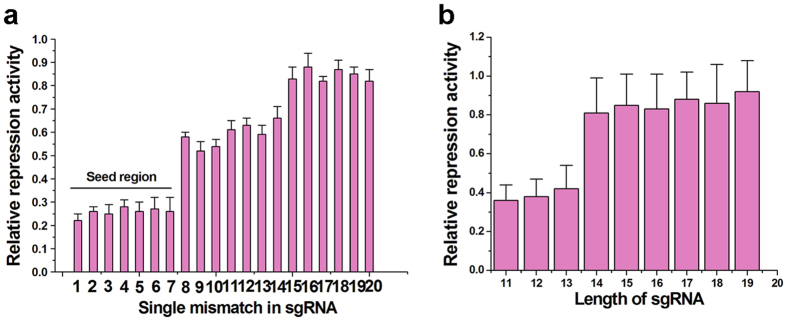
Characterization of the mismatches that influence silencing efficiency. (**a**) The relative effects of repression induced by mutant sgRNAs. The first 7 nt in 5′end of sgRNA seemed to be important for silencing and constituted a “seed” region. (**b**) The length of the antisense domain of the sgRNA affected repression efficiency. The minimal length of the antisense domain for remaining the repression effect was 14 bp. Reported values were mean ± SD from three independent experiments.

To further determine the importance of the antisense domain of sgRNA, we introduced single mutations into the base-pairing region of sgRNA R1 (Fig.1a and Supplementary Table 2) and tested the silencing effects induced by each mutant in Hela cells. A single mutation of the first 7 nt in the 5′ region of sgRNA significantly decreased repression, suggesting that there is a “seed region” for the effect of sgRNA (Fig. 2a). In contrast, the other mismatches still led to a modest decrease in gene expression. We also altered the length of sgRNA R1 and tested how length affected overall silencing. We found that truncation of several bases within the 3′ end of the sgRNA was well tolerated with little loss of activity (Fig. 2b). The minimal length of the antisense domain needed for effectively gene silencing was 14 bp. These data suggest that the 5′ end of the sgRNA is important for the binding affinity of sgRNA, and that off-target effects maybe induced through inhibition of non-target gene translation.

### Catalytically inactive dead Cas9 effectively suppresses expression of endogenous gene

**Figure 3:**
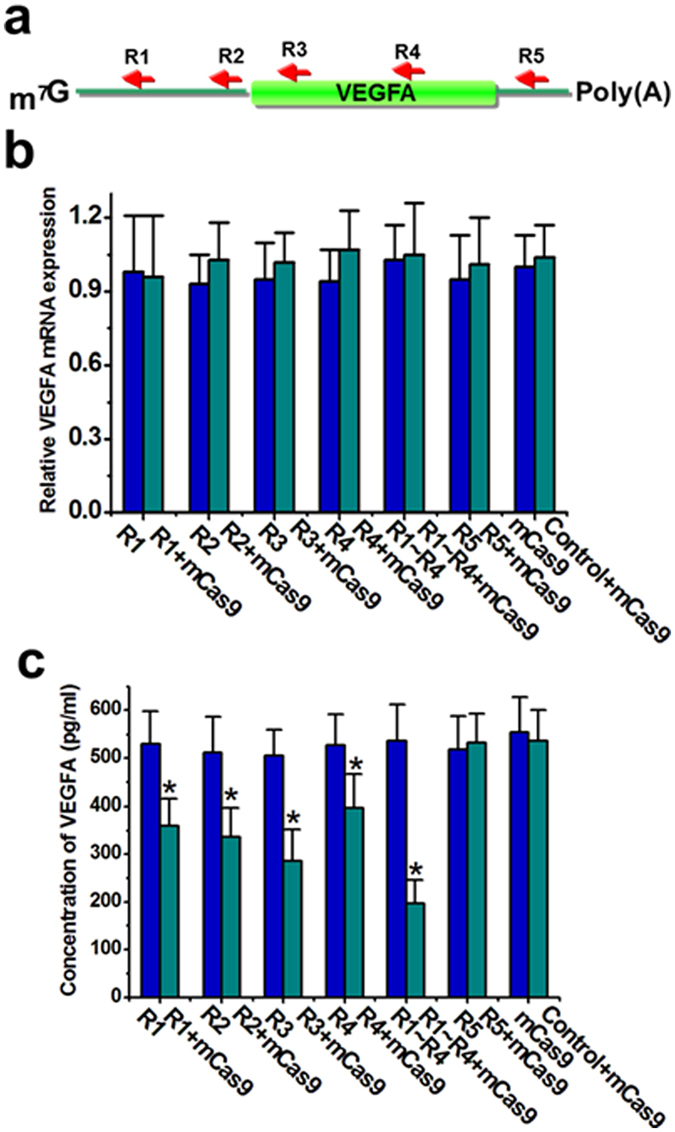
Catalytically inactive dead Cas9 suppressed concentration of VEGFA. (**a**) Design of the sgRNAs targeting various regions of VEGFA mRNA was shown. (**b**) The mutant inactive Cas9 protein had no effect on mRNA expression of VEGFA in the presence of the related sgRNA. Reported data were mean ± SD and the experiments were repeated three times. (**c**) Suppression of VEGFA concentration was achieved by each sgRNA (R1, R2, R3 or R4) combined with the mutant inactive Cas9 protein in Hela cells. Reported data were mean ± SD and the experiments were repeated three times. *P < 0.05 compared to nonspecific sgRNA control by paired, one-sided t-test.

We then determined whether the sgRNA-Cas9 complex could repress translation of endogenous genes in Hela cells. Human vascular endothelial growth factor A (VEGFA) gene encodes a protein that is secreted by cells^25^. Based on the principle above, we designed 5 different sgRNAs (R1, R2, R3, R4 and R5) to target the mRNA of human VEGFA (Fig. 3a and Supplementary Table 3) and used the catalytically inactive mutant Cas9-NLS to test its ability to inhibit gene translation. We confirmed that the subcellular localization for dCas9-NLS is nucleus (Supplementary Fig. 1). As expected, the results of qPCR indicated that the relative levels of VEGF mRNA did not change obviously after transfection of vectors expressing mutant Cas9 and one of the designed sgRNAs (Fig. 3b and Supplementary Table 4). In contrast, all the sgRNAs except R5 decreased the concentration of VEGFA protein to some extent after addition of Cas9 protein and the combination of sgRNAs (R1∼R4) achieved a stronger inhibition effect, as revealed by ELISA assay (Fig. 3c). We then performed an *in vitro* translation reaction and the data demonstrated that mutant Cas9 targeting VEGFA mRNA directly inhibited translation (Supplementary Fig. 3). Furthermore, we also transfected a mutant Cas9 lacking the NLS signal into the Hela cells and observed that the concentration of VEGFA protein was decreased by this Cas9 version (Supplementary Fig. 4). These results further confirmed that the sgRNA-Cas9 complex can repress translation of targeted cellular mRNA and demonstrated that this effect is not related to the nuclease domain of Cas9.

### Evaluate one off-target effect induced by Cas9-mediated inhibition of translation

**Figure 4:**
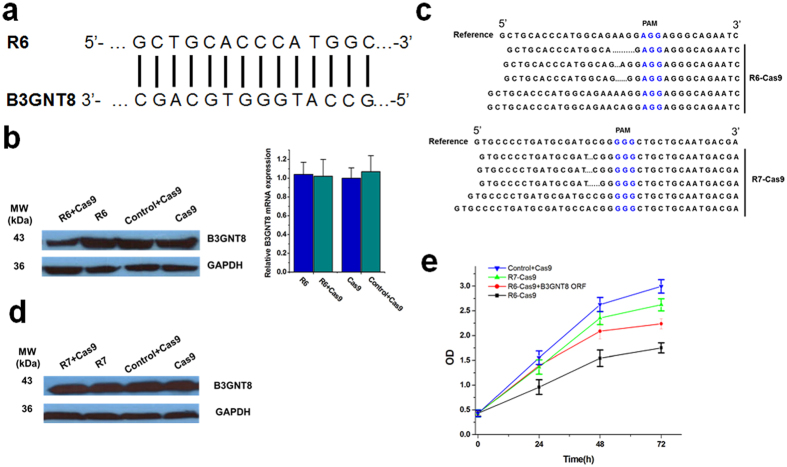
One off-target effect induced by Cas9-mediated inhibition of mRNA translation. (**a**) A potential targeted region of B3GNT8 mRNA that was complementary to the first 14 bases at the 5′ end of R6. (**b**) The result of western-blot indicated that the R6-Cas9 effectively reduces B3GNT8 protein expression. The gels have been run under the same experimental conditions. (**c**) Alignments of VEGFA gene from Hela cells transfected with R6-Cas9 or R7-Cas9. The PAM sequence was indicated in blue. Deletions or insertions to VEGFA DNA caused nonsense mutations. (**d**) The result of western-blot indicated that the expression of B3GNT8 protein was not decreased by R7-Cas9 complex. The gels have been run under the same experimental conditions. (**e**) R6-Cas9 induced an obvious effect in inhibition of cell proliferation (p < 0.01), and B3GNT8 ORF vector partly reversed inhibition of cell proliferation caused by R6-Cas9(p < 0.01). Reported data were mean ± SD and the experiments were repeated three times. ANOVA was used for the comparison of curves of cell proliferation.

After the effects of Cas9 on mRNA translation have been confirmed, we further asked the question whether the sgRNAs designed by conventional method could induce off-target effect through inhibiting mRNA translation of an unintended target gene. We designed two sgRNAs (R6 and R7, also see Supplementary Table 3) to target VEGFA DNA using the online design tool “CRISPR-ERA” (http://CRISPR-ERA.stanford.edu). We then detected one candidate gene that needs to be evaluated for the off-target effects. According to the BLAST search results, B3GNT8 mRNA was found to contain a potential targeting region that is complementary to the first 14 bases at the 5′ end of one designed sgRNA-VEGFA (R6) (Fig. 4a). According to our above results and the previous theory about the seed region of CRISPR, the translation of B3GNT8 mRNA should be blocked by R6-Cas9 complex while the DNA of B3GNT8 may not be cleaved in this case. We then detected the expression of B3GNT8 at both the mRNA and protein level using qPCR (Supplementary Table 4) and western-blot in Hela cells transfected with catalytically active Cas9-NLS and one of the designed sgRNAs (R6 or R7). The results indicated that the R6-Cas9 effectively reduced B3GNT8 expression at the protein level without affecting the level of B3GNT8 mRNA (Fig. 4b). We also confirmed that R6-Cas9 could not disrupt the related sequence of B3GNT8 DNA (Supplementary Table 5). To exclude the possibility that the observed inhibitory effect was induced by knockout of VEGF, another sgRNA-VEGFA (R7) was used to test its effect on B3GNT8 expression. Although both R6-Cas9 and R7-Cas9 caused nonsense mutations at VEGFA and reduced its concentration (Fig. 4c and Supplementary Fig. 5), the results showed that R7-Cas9 complex could not decrease the expression of B3GNT8 at all (Fig. 4d). The frequencies of these sgRNAs-mediated indel mutations at B3GNT8 and VEGFA were shown in Supplementary Table 5. These data strongly suggested that the CRISPR-Cas9 could induce unexpected off-target effects through suppression of unintended target gene translation.

Since B3GNT8 was reported to function as an oncogene in several cancers^26^,^27^, we also determined whether the off-target knockdown effect induced by CRISPR-Cas9 could affect biological behavior of Hela cells derived from cervical cancer. The effect of B3GNT8 on proliferation of Hela cells was investigated using CCK-8 assay. As shown in Fig. 4e, there was a slight difference in cellular proliferation rate between cells in the negative control group and cells transfected with R7-Cas9. The cell viability was found to be significantly reduced after treatment with R6-Cas9, and B3GNT8 ORF vector partly reversed the inhibition of proliferation mediated by R6-Cas9. To further confirm that the observed growth phenotypic changes were caused by knockdown of VEGFA and B3GNT8, we also treated cells with VEGFA or B3GNT8 siRNA and got consistent results (Supplementary Fig. 6). These results indicated that the off-target effects of CRISPR-Cas9 on mRNA translation did indeed cause phenotypic changes.

### The sgRNA-Cas9 complex may repress gene expression through the roadblock mechanism

**Figure 5:**
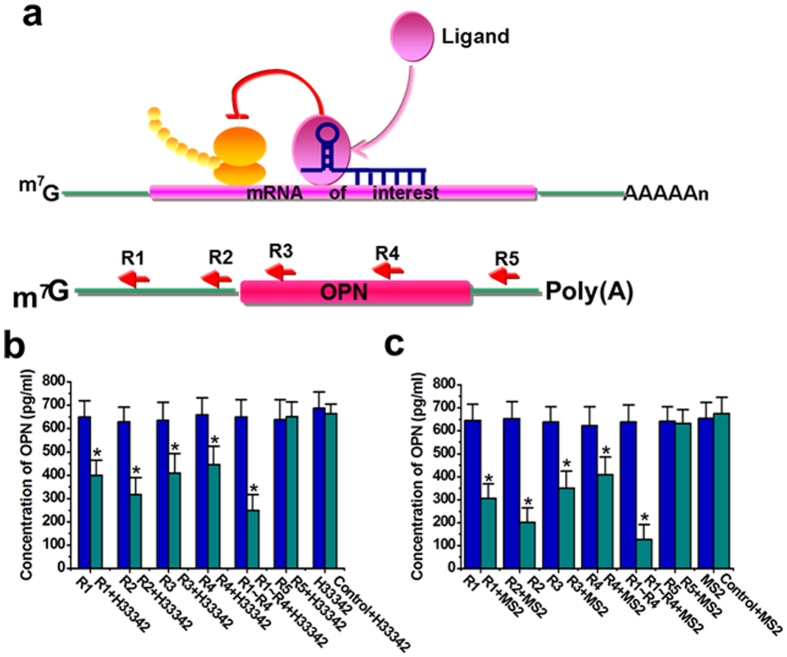
The sgRNA-Cas9 complex repressed gene expression through a universal mechanism. (**a**) The aptamer-ligand complex used the antisense domain to interact with mRNA and suppressed mRNA translation through its function as a roadblock for translation machinery. We designed 5 sgRNAs to target various regions of OPN mRNA. (**b**) Suppression of OPN protein expression in cells by the reprogrammed sgRNAs that bound to 100 uM H33342. Reported data were mean ± SD from three independent experiments. *P < 0.05 compared to nonspecific sgRNA control by paired, one-sided t-test. (**c**) Suppression of OPN protein expression in cells by the reprogrammed sgRNAs that bound to MS2 protein. Reported data were mean ± SD from three independent experiments. *P < 0.05 compared to nonspecific sgRNA control by paired, one-sided t-test.

Since both of the natural and mutant Cas9 proteins can repress translation, we next asked whether there is a general mechanism for explaining this result. RNA aptamers are RNA oligomers capable of binding ligand of choice with high affinity and specificity^28^. In the previous studies, it has been concluded that the aptamer-ligand complex in the 5′-UTR inhibits the translation of downstream gene through a roadblock mechanism^29^,^30^,^31^. Because the sgRNA of CRISPR-Cas9 system also has a Cas9-binding element that functions as an RNA aptamer, we hypothesized that sgRNA-Cas9 complex represses translation through the roadblock mechanism mediated by aptamer-ligand complex (Fig. 5a). If so, another aptamer coupled with a complementary sequence against a target gene should also have the ability to repress the translation of the gene in the presence of ligand. If it is not the case, the repression effect on translation should be contributed to the unknown activity of Cas9 protein. To test this idea, we replaced the Cas9 aptamer in the sgRNA scaffold with the aptamer for Hoechst dye H33342^29^ and designed 5 antisense domains complementary to different regions of the mRNA of osteopontin (OPN). The protein encoded by this gene is a kind of cytokine secreted by cells^32^. As expected, these reprogrammed sgRNAs except R5 (Supplementary Table 6) showed efficient silencing effects on OPN protein expression only in the presence of H33342 (Fig. 5b). The combination of sgRNAs (R1∼R4) also induced a stronger inhibition effect. Relative levels of OPN mRNA did not change obviously between Hela cells harboring the reprogrammed sgRNAs grown in the absence or presence of H33342 (Supplementary Fig. 7 and Supplementary Table 4). To further confirm the effects of this model, we also used the MS2 aptamer for reprogramming the sgRNA^30^. MS2 is the coat protein of a bacteriophage^33^. MS2 overexpression plasmid and vectors expressing one of the reprogrammed sgRNAs (Supplementary Table 7) or expressing the non-target sgRNA control were singly transfected or cotransfected into Hela cells, and ELISA assay revealed that the concentration of OPN was only decreased by one of the reprogrammed sgRNA or the sgRNA combination in the presence of MS2 (Fig. 5c). Similarly, the relative mRNA level of OPN was also not changed by this approach (Supplementary Fig. 8). These results supported our hypothesis that the sgRNA-Cas9 complex may repress mRNA translation through the roadblock mechanism.

## Discussion

In this study, we provided direct evidences to test the hypothesis that Cas9 from S. pyogenes can repress gene expression through inhibition of translation when targeted to RNA sequences that do not have adjacent PAM sequences in the DNA encoding them. Although Cas9 has been clearly shown that it is capable of targeting to a viral RNA and inhibiting translation^24^, we firstly showed that both natural Cas9 and catalytically dead Cas9 can also repress the mRNA translation of cellular genes in the absence of PAM-carrying DNA. We also showed that only the first 14 nt in the 5′ end of sgRNA is essential for this process. While this is in contrast to DNA targeting where the seed region is found at the 3′ end of the spacer^9^, our result is in consistent with the previous findings which suggested that the 5′ end sequence mainly contributes to the substrate binding of siRNAs/shRNAs^34^, indicating a universal principle for RNA-RNA interaction.

The B3GNT8 mRNA containing a region that is complementary to the first 14 bases at the 5′ end of sgRNA-VEGFA was also selected as an example, and the results showed that this unintended target gene can be effectively repressed by the sgRNA-Cas9 complex only at the protein level. Furthermore, we demonstrated that change in B3GNT8 expression has significant effects on cell proliferation. Therefore, undesired biological influences on phenotypes can be occurred through inhibition of unintended target gene translation by CRISPR-Cas9.These findings may limit the capacity of this technology for modification of organism in gene editing, when the off-target mRNA is expressed in the target cell type or developmental stage. The low level of reduction in the proliferation rate of cells transfected with R7-Cas9 was presumably attributed to the knockout effect of VEGFA.

When designing a sgRNA for gene editing or regulation, the specificity is crucial. In the previous studies, it was generally considered that the sgRNA may pair with thousands of other sequences with imperfect matches^35^,^36^,^37^. The researchers have developed some algorithms to predict the potential off-target sequences of one sgRNA in the genome and only the sgRNA with the least number of off-target sites was chosen for further experiments. They also used the truncate sgRNAs to enhance the specificity and examined potential off-target editing in experiments using whole genome sequencing. However, the new discovery revealed by this study suggests that we should also consider the undesired off-target effects induced by inhibition of mRNA translation through searching for mRNA sequences possibly paired with the seed region in 5′ end of sgRNA. Algorithms based on this new seed region theory are still to be developed to further reduce off-target effects.

It is surprising that Cas9 fused to an NLS can effectively suppress gene translation though it should be located in nucleus of the cell. One possible explanation for this phenomenon is that the sgRNA-Cas9 binds the mRNA in the nucleus and remains bound to the mRNA during and after export. This observation is consistent with an earlier study which indicated that mRNA harboring an aptamer can carry the ligand out of the nucleus^38^.

A sgRNA of CRISPR-Cas9 system contains an RNA aptamer for binding to Cas9 protein. Previous work indicated that RNA aptamers are tightened upon binding of a ligand and thereby interfere as an inhibitory complex with the ribosome^29^. Using the designed RNA aptamer-ligand complexes which physically obstruct translation machinery, the results indicated that other large moieties targeted to mRNAs can also inhibit translation. Both sgRNAs and reprogrammed RNA aptamers targeting the 3′-UTR were inefficient for the translational suppression. Based on these observations, we proposed that this Cas9-mediated phenomenon can be explained by roadblock mechanism. Furthermore, these findings significantly broaden the potential uses of the CRISPR technology.

During the course of the peer-review for this work, programmable CRISPR-Cas9 system recognizing endogenous mRNAs were reported^39^. That work demonstrated that mRNA binding by Cas9-sgRNA complex is independent of the PAMmer, and that sgRNA targeting the 3′-UTRs does not influence amount of translated proteins. The authors also indicated that nuclear-localized RNA-targeting Cas9 can be exported to the cytoplasm in the presence of sgRNAs targeting mRNA. Those results were not contrary to our findings in this paper, which revealed that Cas9 containing NLS signal has the ability to interact with mRNA in nucleus and induces a modest suppression of mRNA translation via the roadblock mechanism in cytoplasm. The inhibitory effect of Cas9:sgRNA complex on translation may be further enhanced by the PAMmer oligonucleotide.

In summary, we provide novel advances in understanding the mechanism of action of CRISPR-Cas9 and suggest that the future studies should also detect the potential off-target effects at the protein level. The sgRNA-complex may suppress the translation of many unintended target genes without affecting their DNA sequences. Future works which clearly define this as a broadly applicable phenomenon are still needed. A more broad analysis of the cellular proteome by mass spectrometry may be required.

## Methods

### Plasmids construction

For expressing Cas9 (S. pyogenes) fused to an NLS, the plasmid vectors pSpCas9 (Addgene plasmid #41815) and pdCas9-humanized (Addgene plasmid #44246) were used to express natural and mutant Cas9 protein respectively in cells. For expressing Cas9 not fused to an NLS, the plasmid vectors pcDNA3-CMV-Cas9 and pcDNA3-CMV-dCas9 were used to transiently express natural and mutant Cas9 protein lacking NLS respectively in human cells. The cDNA sequences for sgRNAs with ribozyme sequences at both ends^40^ were designed, synthesized, and inserted into pRL-SV40 vector at restriction site of Hind III/Xba I respectively. To construct plasmid pcDNA3-MS2, cDNA sequence for MS2 gene was inserted into pcDNA3 digested with BamHI/EcoRI. The B3GNT8 ORF containing pReceiver vector was purchased from GeneCopoiea Inc, Rockville, MD, USA.

### Cell culture and cell transfection

Hela cells were purchased from American Type Culture Collection (ATCC) and maintained in DMEM medium supplemented with 10% fetal bovine serum (Invitrogen) in the presence of 5% CO_2_ at 37 °C. For transient transfection experiments, cells were treated with the mixtures of plasmids and Lipofectamine 2000 (Invitrogen) according to manufacturer’s instructions when they reached 70–80% confluency.

### Luciferase reporter assay

At 48 h after transfection, Hela cells were lysed and then luciferase activity was measured by the Renilla Luciferase Assay System (Promega, Madison, WI, USA) according to manufacturer’s instructions. The assays were performed in duplicate and the experiments were repeated three times. To calculate the relative activity of luciferase, the activity of Renilla luciferase was normalized to the activity of firefly luciferase.

### *In vitro* translation reaction

Purified Cas9 protein was incubated with 1 μg sgRNA and 1 μg Renilla luciferase (or VEGFA) mRNA and then the mixture was further incubated with the rabbit reticulocyte lysate (Promega, Madison, WI, USA) according to the manufacturer’s instructions. The activity of *in vitro* translated Renilla luciferase was calculated as described above. The concentration of VEGFA was calculated as described below.

### ELISA assay of VEGFA/OPN concentration

The concentration of VEGFA/OPN protein was measured by ELISA assay according to the manufacturer’s instructions. Briefly, 10^6^/sample cells were harvested and the supernatants of lysates were collected through centrifugation. The target protein is quantified using a colorimetric reaction and the optical density of the end-product was calculated using a microplate reader (Bio-Rad, Hercules, CA). Then the values of optical density were converted to protein concentrations through standard curves.

### RNA extraction and real-time quantitative PCR

Total RNA was isolated from cells transfected with the plasmids using TRIzol (Invitrogen) according to the suggested instructions. The cDNA strand was synthesized from total RNA using RevertAidTM First Strand cDNA Synthesis Kit (Fermentas, Hanover, MD) according to the related instructions. Real time quantitative PCR was performed on an ABI PRISM 7000 Fluorescent Quantitative PCR System (Applied Biosystems, Foster City, CA, USA) using All-in-OneTM qPCR Mix (GeneCopoiea Inc, Rockville, MD). The PCR cycling parameters were as follows: 95 °C for 15 min, followed by 40 cycles of 94 °C for 15 s, 55 °C for 30 s and 72 °C for 30 s.

### Western blot analysis

Cells were washed with PBS and lysed in RIPA buffer. The protein concentration was determined through using the BCA protein assay. Equal amounts of whole protein extract were electrophoresed onto SDS–polyacrylamide gels and then transferred to PVDF membranes (Millipore, Billerica, MA). Samples were blocked with 5% dry milk and incubated over-night with the primary antibodies. Then, the samples were incubated with horseradish peroxidase–conjugated secondary antibody (Amersham, Piscataway, NJ) and immunoblots were developed using Super Signal chemiluminescence reagents (Pierce Chemical Co.).

### Determination of NHEJ-mediated indel mutations

Cells were harvested 3 days after transfection and the genomic DNA was extracted using the QIAamp DNA Blood Mini Kit (QIAGEN) according to the company’s instructions. PCR was then performed to amplify the target regions using the genomic DNA as template. Purified PCR products were cloned into Zero Blunt TOPO vector (Life Technologies), and subjected to Sanger sequencing to estimate NHEJ frequencies.

### CCK-8H assay

Cell proliferation was measured using CCK-8 assay. Cells were seeded in a 96-well plate and cultured in medium. At 0, 24, 48 and 72 h after transfection, the cells were added with 10 ml of CCK-8 reagent and incubated at room temperature for an hour. The absorbance in each well was measured by a microplate reader (Bio-Rad, Hercules, CA, USA).

### Statistical analysis

Values are expressed as means ± SD and the statistical analyses were carried out by using software SPSS (Version 17.0). P values were two-sided and a value of < 0.05 was considered to be statistically significant.

## Additional Information

**How to cite this article**: Liu, Y. *et al*. Targeting cellular mRNAs translation by CRISPR-Cas9. *Sci. Rep.* **6**, 29652; doi: 10.1038/srep29652 (2016).

## Supplementary Material

Supplementary Information
