# Supplementary material for: Targeting cellular mRNAs translation by CRISPR-Cas9
Source: Sci Rep. 2016 Jul 13;6:29652. doi: 10.1038/srep29652 (PMC4942795; doi:10.1038/srep29652)
Supplement: Supplementary Information [file srep29652-s1.pdf]

# Targeting cellular mRNAs translation by CRISPR-Cas9

Yuchen Liu [1,2#], Zhicong Chen[1,2#], Anbang He[1,2#], Yonghao Zhan [1,2], Jianfa Li[1,2], Li Liu [1,2], Hanwei Wu [1], Chengle Zhuang [1,2], Junhao Lin [1,2], Qiaoxia Zhang[1], Weiren Huang [1*],

1 Key Laboratory of Medical Reprogramming Technology, Shenzhen Second People's Hospital, First Affiliated Hospital of Shenzhen University, Shenzhen 518039, China

2 Department of Urological Surgery, Shenzhen Second People's Hospital, The First Affiliated Hospital of Shenzhen University, Shenzhen 518039, China

# These authors contributed equally to this work.

* To whom correspondence should be addressed. Weiren Huang, email: pony8980@163.com

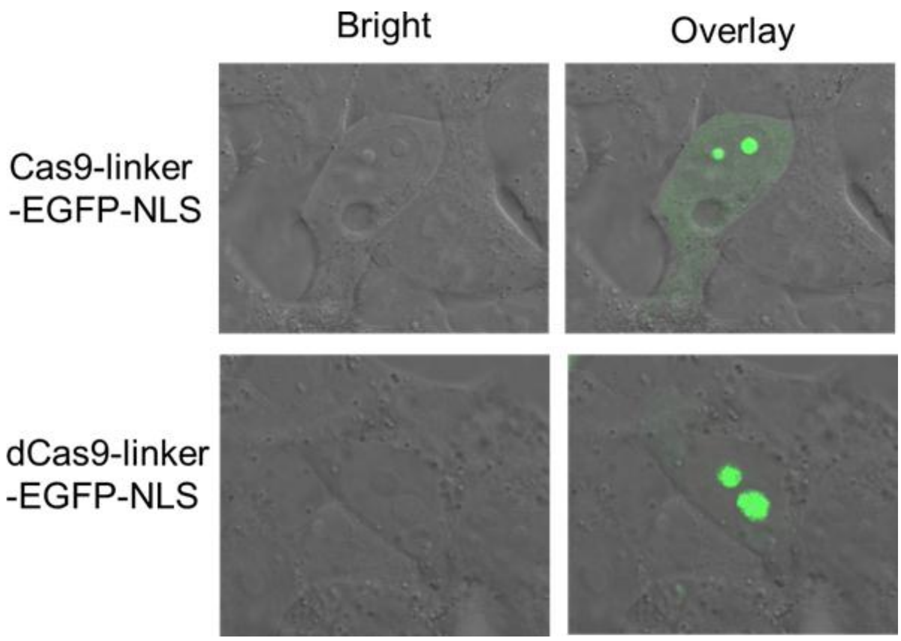


**Supplementary Figure 1.** The subcellular localization for Cas9-GFP-NLS and dCas9-GFP-NLS proteins is nucleus. To determine the subcellular localization of Cas9 or dCas9 protein mediated by the NLS signal, confocal laser-scanning microscope observations were performed 48 h after transient transfection of Cas9-linker-EGFP-NLS or dCas9-linker-EGFP-NLS plasmids. NLS can be used to locate the fusion proteins Cas9-linker-EGFP and dCas9-linker-EGFP to the nucleus.

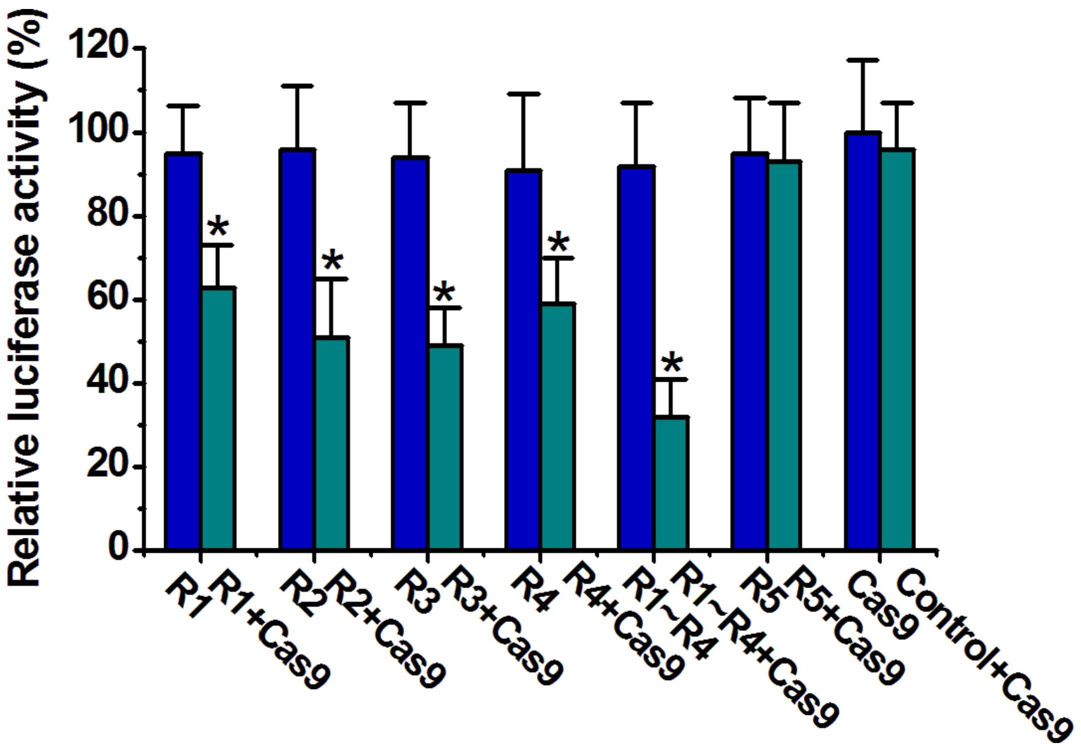


**Supplementary Figure 2.** Cas9 lacking the NLS effectively suppressed activity of luciferase. Suppression of luciferase expression was achieved by each sgRNA (R1, R2, R3 or R4) combined with wild-type Cas9 protein in Hela cells. Introduction of either the sgRNA or Cas9 alone had no such effect, nor did introduction of a nonspecific sgRNA control and Cas9. Reported data are shown as mean ± SD from three biological replicates. *P < 0.05 compared to nonspecific sgRNA control by paired, one-sided t-test.

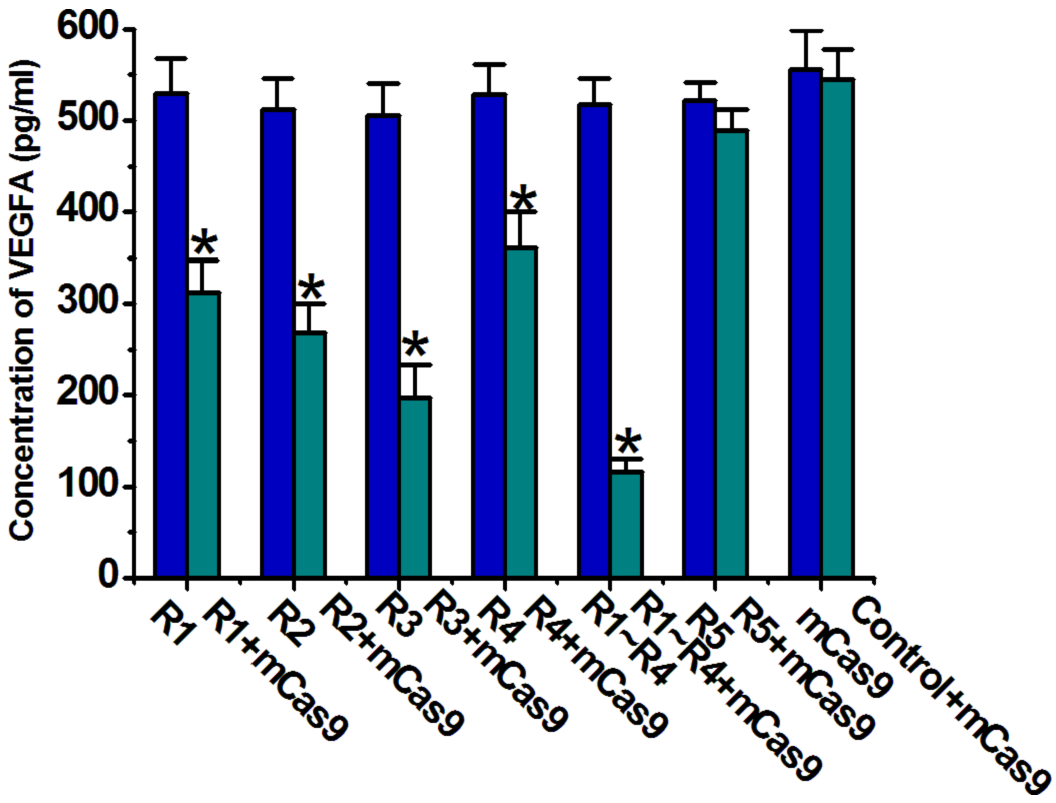


**Supplementary Figure 3.** Mutant Cas9 suppressed in vitro expression of VEGFA. All the sgRNAs except R5 decreased the expression of VEGFA after addition of mutant Cas9 protein. The combination of sgRNAs (R1~ R4) achieved a stronger inhibition effect on VEGFA expression. Reported data were mean ± SD from three biological replicates. *P < 0.05 compared to nonspecific sgRNA control by paired, one-sided t-test.

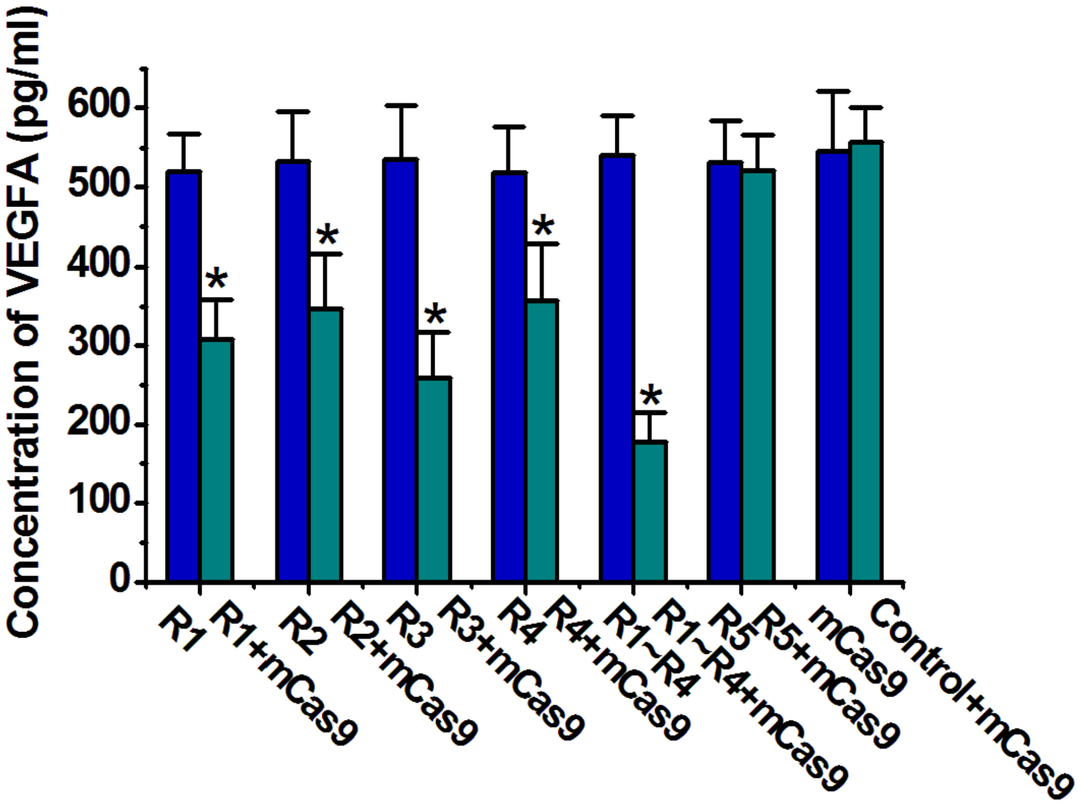


**Supplementary Figure 4.**Mutant Cas9 lacking the NLS signal effectively suppressed VEGFA expression. All the sgRNAs except R5 decreased the concentration of VEGFA after addition of mutant Cas9 protein. The combination of sgRNAs (R1~ R4) achieved a stronger inhibition effect on VEGFA expression. Reported data were mean ± SD from three biological replicates. *$P < 0.05$ compared to nonspecific sgRNA control by paired, one-sided t-test.

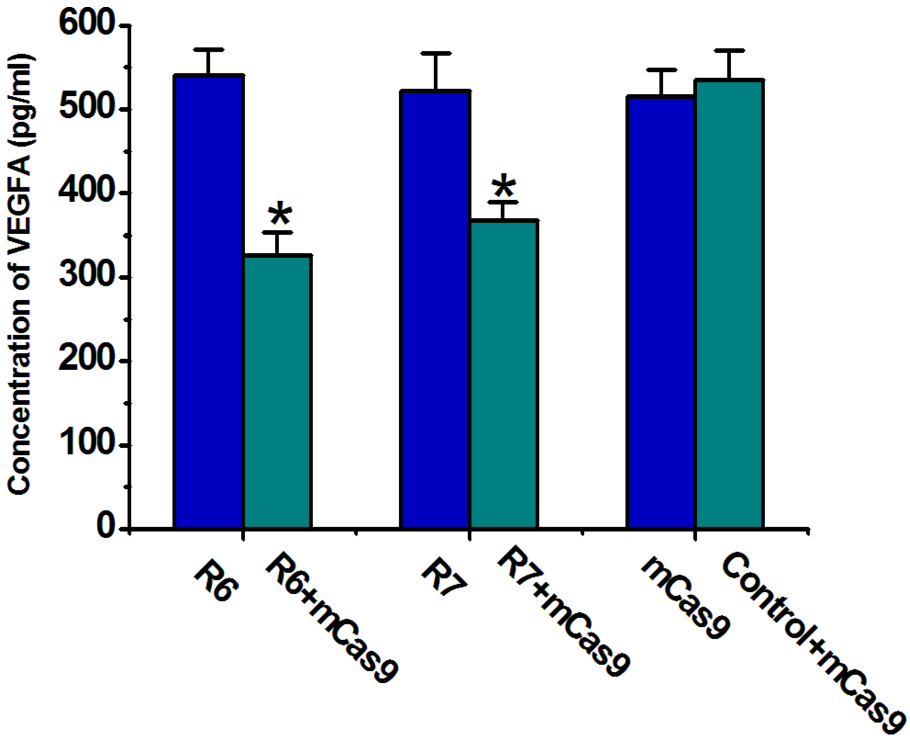


**Supplementary Figure 5.** R6-Cas9 and R7-Cas9 complexes suppressed concentration of VEGFA. Reported data were mean ± SD from three biological replicates. *P < 0.05 compared to nonspecific sgRNA control by paired, one-sided t-test.

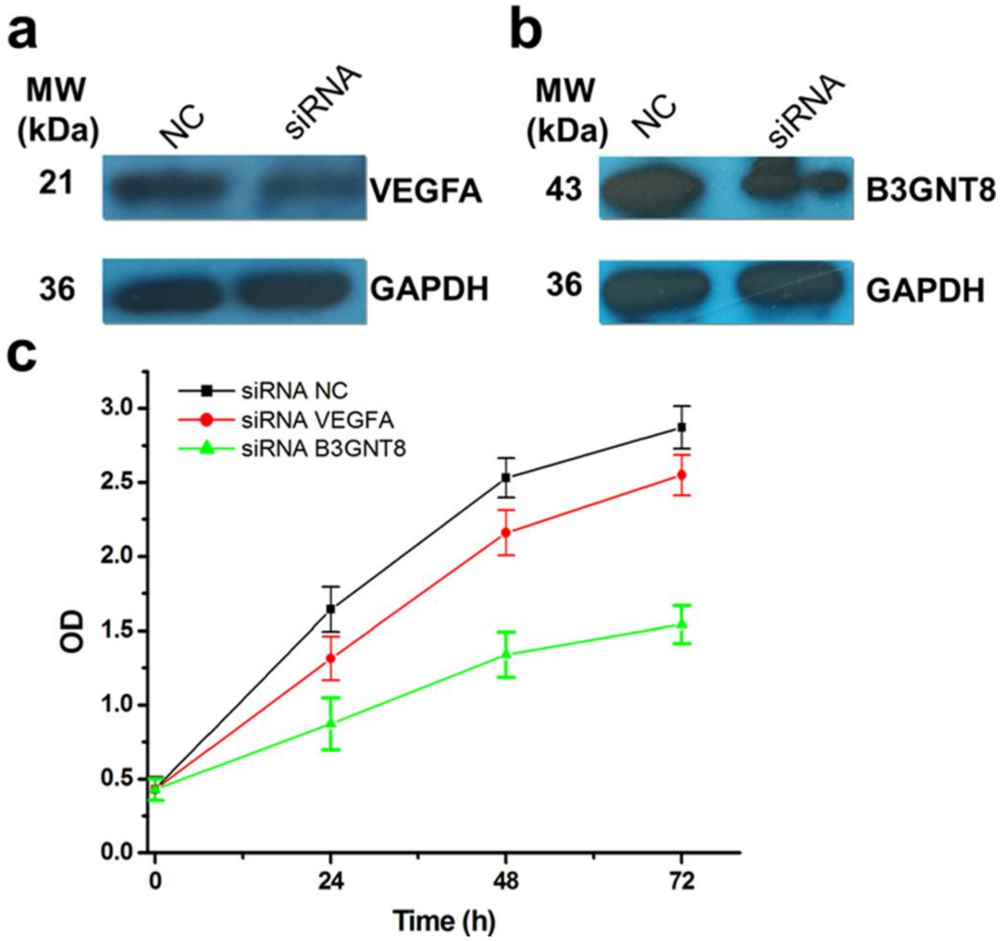


**Supplementary Figure 6.** The effects of siRNAs for VEGFA and B3GNT8 on cell growth. (**a**) The result of western-blot indicated that the expression of VEGFA protein was decreased by its siRNA. The gels have been run under the same experimental conditions. (**b**) The result of western-blot indicated that the expression of B3GNT8 protein was decreased by its siRNA. The gels have been run under the same experimental conditions. (**c**) siRNA-B3GNT8 demonstrated an obvious inhibitory effect on cell proliferation, and siRNA-VEGFA also induced a moderate decrease in cell proliferation. Reported data were mean ± SD and the experiments were repeated three times.

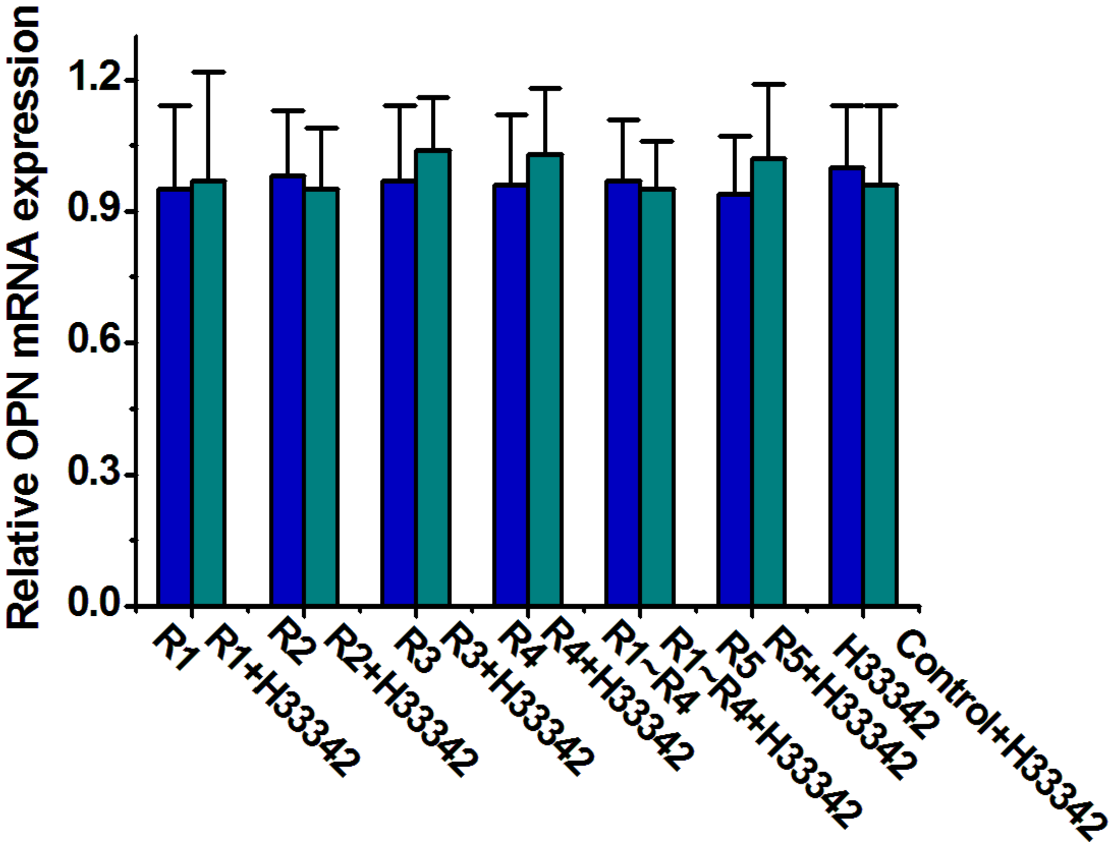


**Supplementary Figure 7.** Relative expression level of OPN mRNA influenced by sgRNA-H33342 complex. The expression level of OPN mRNA was determined by quantitative real-time PCR. GAPDH was used as the internal control. Relative level of OPN mRNA was not changed by the reprogrammed in the absence or presence of H33342. Reported values were presented as mean ± SD and the experiments were repeated three times.

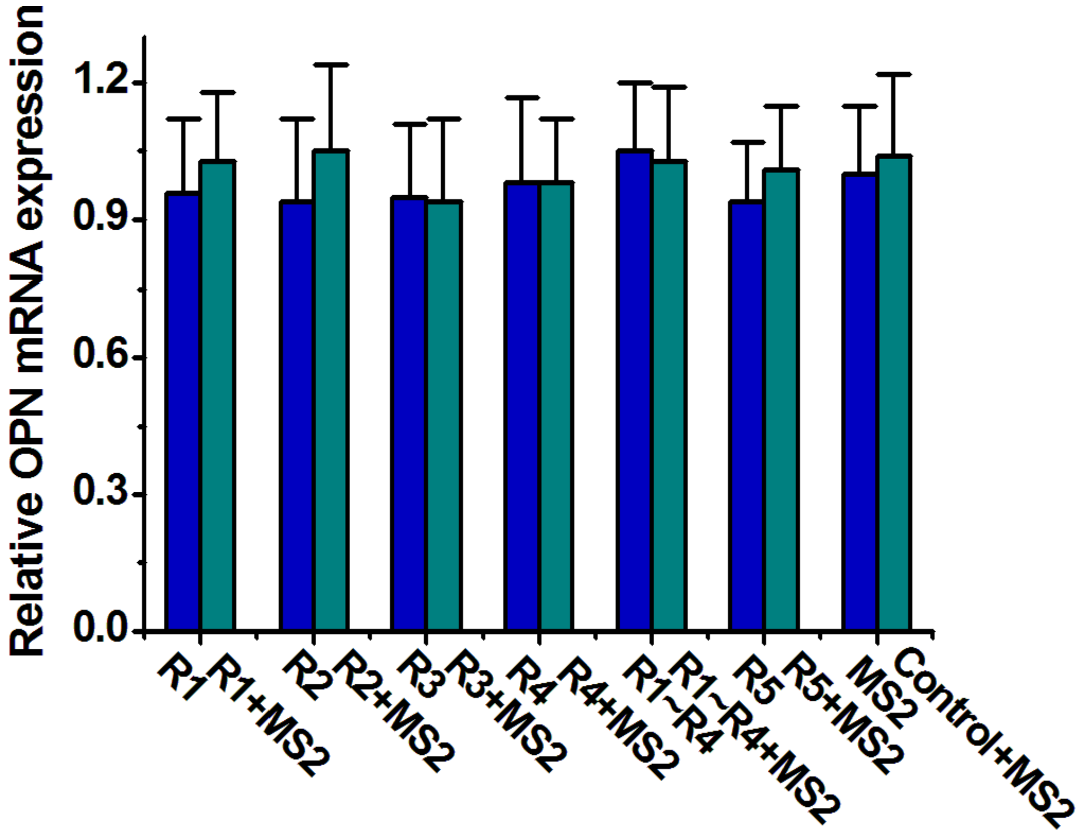


**Supplementary Figure 8.** Relative expression level of OPN mRNA influenced by sgRNA-MS2 complex. The expression level of OPN mRNA was determined by quantitative real-time PCR. GAPDH was used as the internal control. Relative level of OPN mRNA was not changed by the reprogrammed in the absence or presence of MS2. Reported values were presented as mean ± SD and the experiments were repeated three times.

Fig.4b

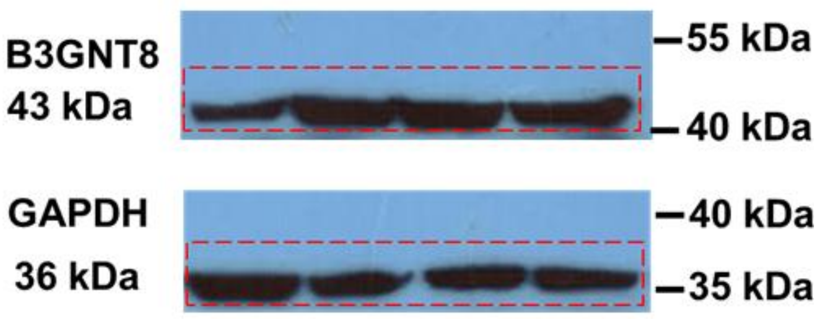


Fig.4c

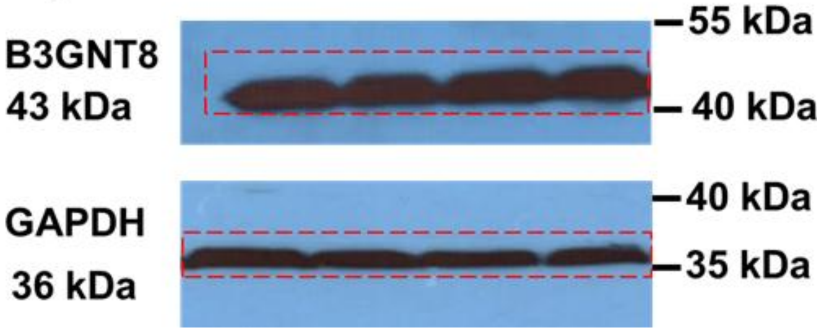


**Supplementary Figure 9.** Uncropped blots of western blot results presented in the main paper.

**Supplementary Table 1.** cDNA sequences of the sgRNAs targeting Renilla luciferase mRNA.

| Names | Sequences |
| --- | --- |
| R1 | TATTGGTCTCCTTAAACCTGGTTTTAGAGCTAGAAATAGCAAG TTAAAATAAGGCTAGTCCGTTATCAACTTGAAAAAGTGGCA CCGAGTCGGTGC |
| R2 | AGAAAGGCAAAGTGGATGTCGTTTTAGAGCTAGAAATAGCAAG TTAAAATAAGGCTAGTCCGTTATCAACTTGAAAAAGTGGCA CCGAGTCGGTGC |
| R3 | CACGTTCATTTGCTTGCAGCGTTTTAGAGCTAGAAATAGCAAG TTAAAATAAGGCTAGTCCGTTATCAACTTGAAAAAGTGGCA CCGAGTCGGTGC |
| R4 | CTTAACGAGAGGGATCTCGCGTTTTAGAGCTAGAAATAGCAAG TTAAAATAAGGCTAGTCCGTTATCAACTTGAAAAAGTGGCA CCGAGTCGGTGC |
| R5 | GGTTTAAACGAATTCCCGGGGTTTTAGAGCTAGAAATAGCAAG TTAAAATAAGGCTAGTCCGTTATCAACTTGAAAAAGTGGCA CCGAGTCGGTGC |

**Supplementary Table 2.** cDNA sequences of the first 20 nt regions of sgRNA R1 mutants used in Fig.2a.

| Mutation Sites | Sequences |
|---|---|
| 1 | **A**ATTGGTCTCCTTAAACCTG |
| 2 | T**T**TTGGTCTCCTTAAACCTG |
| 3 | TA**A**TGGTCTCCTTAAACCTG |
| 4 | TAT**A**GGTCTCCTTAAACCTG |
| 5 | TATT**C**GTCTCCTTAAACCTG |
| 6 | TATTG**C**TCTCCTTAAACCTG |
| 7 | TATTGG**A**CTCCTTAAACCTG |
| 8 | TATTGGT**G**TCCTTAAACCTG |
| 9 | TATTGGTC**A**CCTTAAACCTG |
| 10 | TATTGGTCT**G**CTTAAACCTG |
| 11 | TATTGGTCTC**G**TTAAACCTG |
| 12 | TATTGGTCTCC**A**TAAACCTG |
| 13 | TATTGGTCTCCT**A**AAACCTG |
| 14 | TATTGGTCTCCTT**T**AACCTG |
| 15 | TATTGGTCTCCTTA**T**ACCTG |
| 16 | TATTGGTCTCCTTAA**T**CCTG |
| 17 | TATTGGTCTCCTTAAA**G**CTG |
| 18 | TATTGGTCTCCTTAAAC**G**TG |
| 19 | TATTGGTCTCCTTAAACC**A**G |

| | |
|---|---|
| 20 | TATTGGTCTCCTTAAACCT**C** |

The mutated bases of the sgRNA were highlighted in bold.

**Supplementary Table 3.** cDNA sequences of the sgRNAs targeting VEGFA mRNA or DNA.

| Names | Sequences |
|---|---|
| R1 | TAAATTAAAACGAGAAACAAGTTTTAGAGCTAGAAATAGCAAG TTAAAATAAGGCTAGTCCGTTATCAACTTGAAAAAGTGGCA CCGAGTCGGTGC |
| R2 | CTCCTCTTCCTTCTCTTCTTGTTTTAGAGCTAGAAATAGCAAG TTAAAATAAGGCTAGTCCGTTATCAACTTGAAAAAGTGGCA CCGAGTCGGTGC |
| R3 | TGATGATTCTGCCCTCCTCCGTTTTAGAGCTAGAAATAGCAAG TTAAAATAAGGCTAGTCCGTTATCAACTTGAAAAAGTGGCA CCGAGTCGGTGC |
| R4 | TTGGTCTGCATTCACATTTGGTTTTAGAGCTAGAAATAGCAAG TTAAAATAAGGCTAGTCCGTTATCAACTTGAAAAAGTGGCA CCGAGTCGGTGC |
| R5 | GCTGCACCCATGGCAGAAGGGTTTTAGAGCTAGAAATAGCAAG TTAAAATAAGGCTAGTCCGTTATCAACTTGAAAAAGTGGCA CCGAGTCGGTGC |
| R6 | GTGCCCCTGATGCGATGCGGGTTTTAGAGCTAGAAATAGCAAG TTAAAATAAGGCTAGTCCGTTATCAACTTGAAAAAGTGGCA CCGAGTCGGTGC |
| R7 | GAAACCCTGAGGGAGGCTCCGTTTTAGAGCTAGAAATAGCAAG TTAAAATAAGGCTAGTCCGTTATCAACTTGAAAAAGTGGCA CCGAGTCGGTGC |

**Supplementary Table 4**. Primer sequences used in real-time quantitative PCR.

| Names | Sequences |
| --- | --- |
| VEGF-F | ACAGACACCGCTCCTAGCCC |
| VEGF-R | CGAGAACAGCCCAGAAGTTGG |
| β3GnT8-F | CCCTGACTTCGCCTCCTAC |
| β3GnT8-R | GGTCTTTGAGCGTTCGGTTGA |
| OPN-F | AATGGTGCATACAAGGCCATC |
| OPN-R | TGTCCTTCCCACGGCTGT |
| GAPDH-F | CGCTCTCTGCTCCTCCTGTTC |
| GAPDH-R | ATCCGTTGACTCCGACCTTCAC |

**Supplementary Table 5.** Frequencies of the sgRNAs-mediated indel mutations at B3GNT8 and VEGFA.

| Genes | sgRNAs | Indel mutation frequency (%) |
|---|---|---|
| **B3GNT3** | **R6** | **N.D.** |
| | **R7** | **N.D.** |
| **VEGFA** | **R6** | **25.8±3.1** |
| | **R7** | **22.3±2.9** |

Mean indel frequencies in Hela cells were determined using the method described in Methods Section. Data were shown as mean±s.e.m. N.D., none detected.

**Supplementary Table 6.** cDNA sequences of the reprogrammed sgRNAs used in Fig.5b.

| Names | Sequences |
|---|---|
| R1 | TGCAACTGGCCTGAGACGAGGGTGATCAGATTCTGATCCAATGTTATGCTTCTCTGCCTGGGAACAGCTGCCTGAAGCTTTGGATCCGTCGC |
| R2 | GTAGTGAGTTTTCCTTGGTCGGTGATCAGATTCTGATCCAATGTTATGCTTCTCTGCCTGGGAACAGCTGCCTGAAGCTTTGGATCCGTCGC |
| R3 | TAACTGGTATGGCACAGGTGGGTGATCAGATTCTGATCCAATGTTATGCTTCTCTGCCTGGGAACAGCTGCCTGAAGCTTTGGATCCGTCGC |
| R4 | TTTAATTGACCTCAGAAGATGGTGATCAGATTCTGATCCAATGTTATGCTTCTCTGCCTGGGAACAGCTGCCTGAAGCTTTGGATCCGTCGC |
| R5 | AAGAAGCATTTCATGTTCTCGGTGATCAGATTCTGATCCAATGTTATGCTTCTCTGCCTGGGAACAGCTGCCTGAAGCTTTGGATCCGTCGC |

**Supplementary Table 7.** cDNA sequences of the reprogrammed sgRNAs used in Fig.5c.

| Names | Sequences |
|---|---|
| R1 | TGCAACTGGCCTGAGACGAGCGTACACCATCAGGGTACG |
| R2 | GTAGTGAGTTTTCCTTGGTCCGTACACCATCAGGGTACG |
| R3 | TAACTGGTATGGCACAGGTGCGTACACCATCAGGGTACG |
| R4 | TTTAATTGACCTCAGAAGATCGTACACCATCAGGGTACG |
| R5 | AAGAAGCATTTCATGTTCTCCGTACACCATCAGGGTACG |
